# Pharmacokinetic and Pharmacodynamic Considerations in Antimalarial Dose Optimization

**DOI:** 10.1128/AAC.00287-13

**Published:** 2013-12

**Authors:** Nicholas J. White

**Affiliations:** Mahidol-Oxford Tropical Medicine Research Unit, Faculty of Tropical Medicine, Mahidol University, Bangkok, ThailandCentre for Tropical Medicine, Churchill Hospital, Oxford University, Oxford, United Kingdom

## Abstract

Antimalarial drugs have usually been first deployed in areas of malaria endemicity at doses which were too low, particularly for high-risk groups such as young children and pregnant women. This may accelerate the emergence and spread of resistance, thereby shortening the useful life of the drug, but it is an inevitable consequence of the current imprecise method of dose finding. An alternative approach to dose finding is suggested in which phase 2 studies concentrate initially on pharmacokinetic-pharmacodynamic (PK-PD) characterization and *in vivo* calibration of *in vitro* susceptibility information. PD assessment is facilitated in malaria because serial parasite densities are readily assessed by microscopy, and at low densities by quantitative PCR, so that initial therapeutic responses can be quantitated accurately. If the *in vivo* MIC could be characterized early in phase 2 studies, it would provide a sound basis for the choice of dose in all target populations in subsequent combination treatments. Population PK assessments in phase 2b and phase 3 studies which characterize PK differences between different age groups, clinical disease states, and human populations can then be combined with the PK-PD observations to provide a sound evidence base for dose recommendations in different target groups.

## INTRODUCTION

The primary objective of treating severe malaria is to save life. Other considerations such as preventing recrudescence or minor toxicity are secondary. In uncomplicated malaria, the main objective of antimalarial drug treatment is cure of the infection. Speed of response is also important, as this reflects the rate at which the disease is controlled and the corresponding reduction in the risk of progression to severe malaria. Less-serious adverse effects therefore become a more important factor in determining dose. The therapeutic response in malaria is determined by the concentration profile (pharmacokinetics [PK]) of active antimalarial drug or drugs in the blood (as the asexual parasites which cause malaria pathology are confined to the blood), their intrinsic pharmacodynamic (PD) properties, the susceptibility of the infecting parasites to the drug(s), the number of asexual malaria parasites in the blood, and the activity of host-defense mechanisms. Ideally, antimalarial treatment should be 100% effective in everyone, but this may not be possible without producing toxicity or recommending a long course of treatment with consequent poor adherence. It is now recommended that all antimalarial treatments for uncomplicated malaria should aim at a >95% cure rate for the blood-stage infection (1). In recent years, a general agreement has been reached on methods of clinical and parasitological assessment to measure the cure rates in cases of uncomplicated falciparum malaria (1–3). In Plasmodium vivax and *P. ovale* infections, persistent liver-stage parasites (hypnozoites) cause later relapses, despite cure of the blood-stage infection, which complicates therapeutic assessment. These infections require additional treatment with 8-aminoquinolines (radical cure). Relapses are often genetically heterologous and cannot be distinguished reliably from recrudescences or new infections. This necessitates a different approach for assessment of treatment efficacy in the relapsing malarias—which is yet to be agreed upon.

Many of the antimalarial drugs in current use were introduced at suboptimal doses. For various reasons, quinine, sulfadoxine-pyrimethamine, primaquine (for radical cure of tropical frequent relapsing P. vivax infections), mefloquine, halofantrine, artemisinin derivatives, artemether-lumefantrine, and dihydroartemisinin-piperaquine (i.e., 7 of the 12 current antimalarials) were all deployed initially at doses which were too low in some or all age groups. Pyrimethamine and sulfadoxine doses for children were extrapolated from experience in Caucasian and Asian adults. Their pharmacokinetic properties were not studied in younger age groups before widespread deployment in Africa, where children are the main target group (4). The dose was too low in young children. The primaquine dose regimen (15 mg base/day adult dose) was developed largely on the basis of studies of the long-latency Korean vivax malaria, but this dose was then recommended widely in areas with the more resistant tropical relapse P. vivax phenotypes (5). In Southeast Asia and Oceania, this dose is too low. Five-day primaquine regimens were deployed very widely for radical cure of vivax malaria for over 30 years—yet these regimens were largely ineffective. Fourteen-day courses are now recommended. Mefloquine was first introduced at a single dose of 15 mg base/kg of body weight (6, 7), which may have hastened the emergence of resistance (8). The total dose now recommended is 25 mg/kg divided over 2 or 3 days. The doses of artemisinin derivatives used initially as monotherapy, and then subsequently in combination treatments (artemether at 1.6 mg/kg/dose in artemether-lumefantrine and dihydroartemisinin at 2.5 mg/kg/dose together with piperaquine), may not provide maximal effects in all patients. The initial treatment regimen of artemether-lumefantrine deployed was a four-dose regimen which provided insufficient lumefantrine and gave high failure rates (six doses are now recommended) (9). The dose of dihydroartemisinin in the first formulations of the dihydroartemisinin-piperaquine combination was <2 mg/kg (it is now 2.5 mg/kg, which may still be too low) (10). The pharmacokinetic properties of piperaquine are different in children from in adults, and there is evidence that current dosing schedules in children may be suboptimal (11). After 3 centuries of reasonable dosing based originally upon the Schedula Romana, treatment recommendations for quinine in severe malaria were suddenly reduced in the 1970s to a dose as low as 5 mg/kg/24 h, which is eight times lower than that now recommended. In contrast, the quinine loading dose in severe malaria was not introduced until the early 1980s, and it is still not recommended universally (12). The initial recommendation for artesunate treatment in severe cases of malaria was a daily maintenance dose of half the initial dose (1.2 mg/kg). As oral bioavailability is approximately 60%, this corresponds to an oral dose of 2 mg/kg (13, 14). The currently recommended parenteral dose is twice this and is the same as the recommended initial dose, 2.4 mg/kg/day (1, 15, 16). Recent evidence suggests that this dose should be increased in young children (17).

Optimizing drug dosing requires characterization of the pharmacokinetic and pharmacodynamic properties of the drug in the target populations. There are four main determinants of the therapeutic response: antimalarial pharmacokinetics (affected by variables such as coadministration with food, age, pregnancy, disease severity, vital organ dysfunction, partner drug, and coinfections/other drugs), parasite susceptibility (incorporating effects on different stages of asexual parasite development, dormancy, propensity for resistance to develop, and level of resistance first selected), host defense (influenced by age, pregnancy, and transmission intensity/exposure history), and parasite burden. In addition, mixed infections can be a factor. For antimalarials, *ex vivo* systems are useful for predicting resistance (18) and they provide valuable pharmacodynamic information (19), but they are simply not good enough yet to replace *in vivo* evaluations for dose finding. In uncomplicated falciparum malaria, it is generally agreed that combinations, preferably, fixed-dose combinations (FDC), should be used. The same should apply to vivax malaria, although chloroquine and primaquine can be considered a combination. When drugs are first developed, there is a limited window of opportunity to define the dose-response (or concentration-effect) relationship for the single new compound, but this opportunity must be taken (20). Once the drug is available only as an FDC, the dose ratio is, by definition, fixed and it is too late for optimization of the individual component doses. Characterizing the individual drug dose-response relationships is essential for rational dose optimization, and so a good drug development approach involves documenting the blood concentrations that are associated with submaximal antimalarial effects. Studies in animal models, particularly with P. falciparum, may be informative, but studies in humans will also be needed. It is important to accept that this may result in temporary therapeutic failures in some volunteers. There is a natural reluctance to accept this, but sensitive detection methods to measure low parasite densities now provide us with safe methods that should avoid any risk or discomfort to the patient (21). Suggestions are provided here for an alternative PK-PD approach for dose finding which, if validated, may improve and accelerate dose finding and so avoid systematic underprescribing and thus underdosing. It might also prove more rapid and less expensive. The primary objective is determination of the *in vivo* MIC as the basis for rational dosing (the MIC is the concentration at which the parasite multiplication factor per asexual cycle is 1). It is necessary first to consider the factors which affect the pharmacokinetic properties of antimalarials in malaria and then to consider antimalarial pharmacodynamics and how PK-PD relationships should be assessed.

## PHARMACOKINETICS

**Fig 1:**
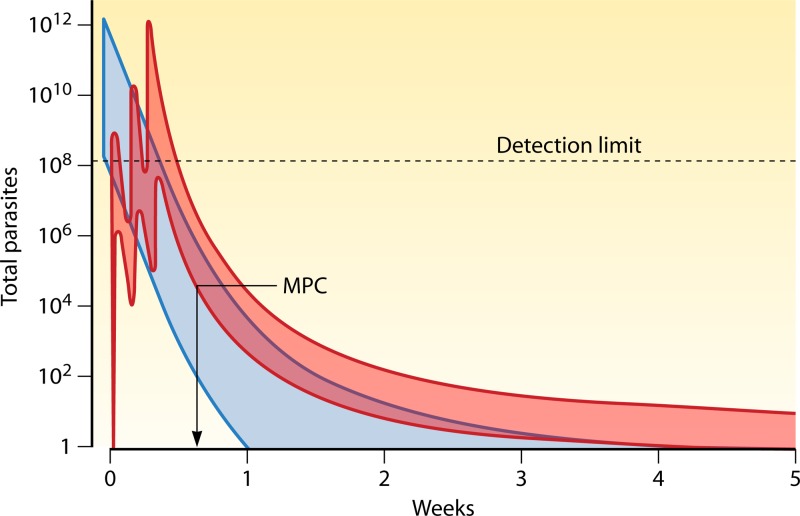
Population PK-PD responses following a 3-day treatment with a hypothetical slowly eliminated antimalarial drug. The total numbers of malaria parasites in the body over time are depicted in blue in a range of patients presenting with parasite densities between approximately 50 and 200,000/μl. The ranges of drug concentration profiles are shown in red, with the corresponding ranges of parasitological responses in blue. Parasitemia levels cannot be counted reliably by microscopy below 50/μl (corresponding to ∼100,000,000 parasites in the body of an adult). The MPC is the lowest blood, plasma, or free plasma concentration which produces the maximum parasiticidal effect (i.e., the maximum parasite reduction ratio). This corresponds to the concentration associated with first slowing of the first-order (log-linear) decline in parasitemia.

The pharmacokinetic (PK) properties of antimalarial drugs are often altered in patients with malaria compared with healthy subjects. The PK properties therefore change as the patient recovers. PK properties are also often significantly different in important patient subgroups such as young children and pregnant women (22). Several of the antimalarial drugs, notably those which are hydrophobic and lipophilic, are poorly absorbed after oral or intramuscular administration and show wide interindividual differences in concentration profiles. In general, this variation in blood concentrations is inversely proportional to bioavailability, which emphasizes the importance of improving bioavailability in drug development. Increasing bioavailability provides the twin benefits of reducing the required dose and thus the cost of the drugs and reducing the individual probabilities of underdosing or overdosing. In considering antimalarial dosing in the past, we tended to concentrate on mean or median values of PK variables, but it is the patients with the lowest blood concentrations who are most likely to fail treatment and facilitate the emergence of resistance and those with the highest concentrations who are most likely to experience drug toxicity (23). These extremes need to be defined, which means that characterizing the distributions of PK variables in important target groups is as important as assessing their measures of central tendency (Fig. 1). Characterizing these distributions well eventually requires sampling of relatively large numbers of patients, which in turn usually necessitates sparse sampling and population PK modeling. Optimal design approaches can be used to ensure that the information is gathered most efficiently (24). It is essential that key patient groups such as young children and pregnant women are studied specifically, and there should be a postregistration commitment to this if such investigations have not been conducted during preregistration studies. There may also be clinically relevant pharmacogenetic differences in drug metabolism between different ethnic groups. Thus, characterizing the distributions of pharmacokinetic variables is a gradual process accrued during phase 2 and phase 3 of drug development, but it must continue into phase 4 to cover all relevant populations.

Malaria is often worst in remote rural areas. The recent development of simple methodologies such as drug measurement from capillary blood filter paper samples (25, 26) will facilitate community-based assessments in remote settings and make sampling of infants and children feasible. Thus, population PK information will eventually be needed in all important target groups (i.e., infants, children, pregnant women, lactating women, malnourished patients, patients receiving antituberculosis [anti-TB] and antiretroviral drugs, etc.) (22) to provide optimal dose recommendations. There is currently limited bioanalytical capacity to support such studies, but there are international schemes to assist antimalarial drug measurement and ensure the accuracy of the results, which should facilitate future laboratory bioanalytical capacity development in tropical countries (27, 28).

In drug development, where a new compound has not been used previously, there is little information on distributions of PK variables and so the important but difficult issue is to determine how much PK-PD information is enough to decide upon a dosage recommendation. For safety reasons, the PK information is usually gathered in the following standard sequence: experimental animals, healthy normal volunteers, adult patients with uncomplicated malaria, children, and, much later, infants and pregnant women.

## PHARMACODYNAMICS

### (i) Action of the drugs.

The antimalarial drugs differ in their stage specificities of action against malaria parasites. The 8-aminoquinolines are unusual in killing pre-erythrocytic-stage parasites, hypnozoites, and mature gametocytes of P. falciparum but having weak activity against its asexual stages (29). They are more active against asexual stages of P. vivax and P. knowlesi. All other antimalarial drugs in current use kill the asexual and sexual stages of sensitive P. vivax, *P. malariae*, *P. ovale*, and P. knowlesi and the asexual stages and early gametocytes (stages I to III) of sensitive P. falciparum, but they do not kill the mature P. falciparum gametocytes (stage V). The artemisinins have a broader range of effect on developing P. falciparum sexual stages, as they also kill stage IV and younger stage V gametocytes. Atovaquone and the antifols kill preerythrocytic stages and have sporontocidal activity in the mosquito (interfering with oocyst formation and therefore blocking transmission). Apart from the 8-aminoquinolines, none of these drugs have significant effects on P. vivax or *P. ovale* hypnozoites. Even within the asexual cycle there are differences in antimalarial activity in relation to parasite development. None of the currently used drugs have significant effects on very young ring stages or mature schizonts, and all have their greatest effects on mature trophozoites in the middle of the asexual cycle (30). In addition, the artemisinins (and other antimalarial peroxides) have substantial ring-stage activity which underlies their life-saving benefit in treatment of severe falciparum malaria (15, 16, 31). Several antimalarials, notably, some antibiotics with antimalarial activity, have greater effects in the second than in the first drug-exposed asexual cycle (23). The pharmacokinetic-pharmacodynamic relationships (PK-PD) have not been very well characterized for any of these activities.

### (ii) *In vivo* pharmacodynamic measures.

**Fig 2:**
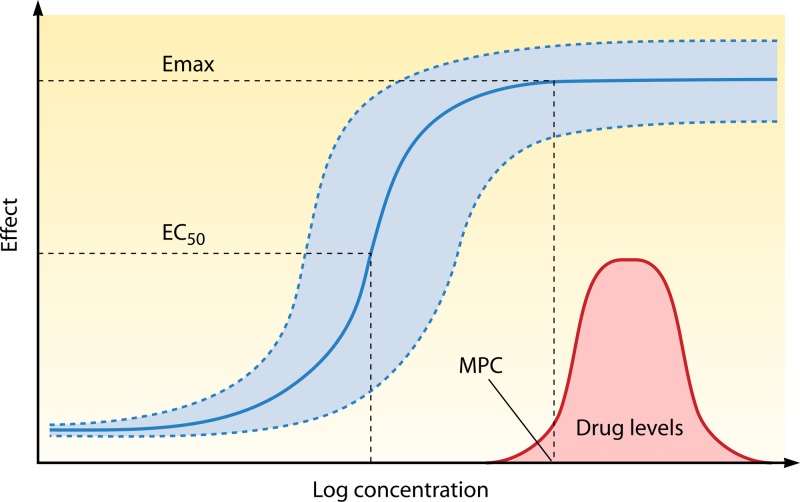
The concentration-effect relationship; for antimalarial drugs, the effect is parasite killing, which can be measured in different ways. The Emax is the maximum parasite killing that a drug can produce, which translates *in vivo* into the maximum parasite reduction ratio. The EC_50_ is the blood or plasma concentration providing 50% of maximum killing. The median and range values for a hypothetical population of malaria parasites are shown in blue, and the distribution of average drug levels in patients is shown as a red bell-shaped curve (i.e., concentrations are log-normally distributed). Clearly, some of the patients have average drug levels below the MPC and would not have maximum responses with this dose regimen.

In severe malaria, the primary therapeutic concern is the speed of parasite killing and, in particular, the killing of circulating ring-stage parasites before they mature and sequester (30, 31). Rapid killing of young P. falciparum parasites by artemisinin and its derivatives explains much of the superiority of artesunate over quinine in the treatment of severe falciparum malaria (15, 16). In uncomplicated malaria, rapid ring-form killing is also important, as it contributes to the speed of patient recovery, but the main therapeutic objective is to reduce parasite multiplication. Once antimalarial treatment is started, then, after a variable lag phase, parasite killing *in vivo* approximates to a first-order process (32–34) as represented by the following equation:

(1) $$P_{t}=P_{0}\;e^{-k_{p^{t}}}$$

where *P_t_* is the parasitemia level at any time *t* after starting treatment, *P*_0_ is the parasitemia level immediately before starting treatment, and *k_p_* is the first-order parasite elimination rate constant. The parasite clearance half-life is therefore 0.693/*k_p_*. In equation 1, parasite killing equates with parasite removal from the circulation, but in falciparum malaria (but not the other malarias) there is an additional major factor removing parasites from the circulation, and that is cytoadherence. Only parasites in the first third of the asexual cycle circulate, and the more mature parasites are sequestered. This complicates interpretation of the parasite clearance curve following treatment with drugs which do not kill ring-form parasites, as initial declines in parasitemia result mainly from sequestration and not drug effects (33). Parasite killing can be expressed as the parasite reduction ratio (PRR), which is the fractional reduction in parasite numbers per asexual cycle, or the reciprocal of ring-form *k_p_* per cycle (32). This cancels out the effects of cytoadherence, as the parasite populations are assessed at the same stages of development separated by one cycle. The shape of the concentration-effect relationship *in vivo* is assumed always to be sigmoid, as it is *in vitro* (Fig. 2), per the following equation:

(2) $$k=k_{\max\;}\cdot [C^{n}/{{EC}_{50}}^{n}+C^{n}]$$

where *k* is the parasite killing rate and *k*_max_ is the maximum parasite killing rate (i.e., the maximum effect, or *E*_max_) for that drug in that infection, *C* is the concentration of drug in blood or plasma, EC_50_ is the blood or plasma concentration resulting in 50% of the maximum effect, and *n* is a parameter defining the steepness of the dose-response relationship. For most drugs, maximum effects are probably achieved initially. The evidence for this is the lack of a relationship between peak concentrations and parasite clearance (the exception is quinine treatment of severe malaria without a loading dose, which provides submaximal effects in some patients) (12). So while concentrations exceed the minimum parasiticidal concentration (MPC), *k_p_* in equation 1 is equal to *k*_max_. It should be noted that each end of the sigmoid curve approaches 0% and 100% effects asymptotically—so the MPC is an approximation, whereas the EC_50_ is a more robust and precise estimate. Once antimalarial concentrations in blood decline to a level below the MPC, the parasite killing rate declines (see the “Antimalarial pharmacokinetic-pharmacodynamic relationships” section below). For drugs in current use, maximum PRRs range from approximately 10-fold to approximately 10,000-fold reductions in parasitemia per asexual cycle. The mean values and their variance *in vivo* have not been established for several important antimalarial drugs in current use (notably lumefantrine and piperaquine), and for others, where monotherapies have been evaluated, the estimates are often imprecise. There is no evidence for saturation of parasite clearance, but, obviously, the higher the initial biomass, the longer it takes to eliminate all the parasites from the body (33). Consequently, patients with high-biomass infections need more antimalarial drug exposure than those with low-biomass infections.

### (ii) *In vitro* susceptibility.

For antimalarial effects, the shape and position of the concentration-effect curve studied *ex vivo* depends on the susceptibility of the infecting parasites and the PD readout (typically, for blood stages, inhibition of growth or maturation, inhibition of hypoxanthine uptake, inhibition of protein or nucleic acid synthesis, etc.). Furthermore, each *in vitro* method assesses a slightly different section of the asexual life cycle, which may result in important differences between methods in the results for drugs with ring-stage activity. It is not clear exactly how the effects of these static drug concentrations in a small volume of dilute blood in the laboratory correspond with *in vivo* effects (18, 19, 35). Neither is the relationship between inhibition of parasite growth and subsequent inhibition of multiplication well established. Inhibition of growth is measured in most *in vitro* tests, whereas in *in vivo* patient studies, inhibition of multiplication (parasite clearance) is recorded. In the absence of *in vivo* information on the concentration-effect relationship, for predictive modeling purposes the slopes of the linear segments of the *in vitro* and *in vivo* sigmoid concentration-effect relationships have been assumed to be similar (8, 35), but whether or not such an assumption is justified remains to be established. Most agree that the antimalarial drug concentration that is biologically relevant in assessing blood-stage effects is the (unbound) fraction in plasma. Total red cell concentrations are less informative as the parasitized red cells behave very differently from their unparasitized counterparts. In the patient, the blood concentrations of the antimalarial drug are changing constantly, and the parasite age distributions may differ considerably between patients. *Ex vivo* systems with changing antimalarial concentrations that are more biologically relevant than the simple static drug susceptibility assays have therefore been developed, and measurement of multiplication inhibition can yield valuable information (19). Rodent models capable of sustaining human malaria infections have also been developed recently (36). Human malaria infections in immunodeficient mice allow PK-PD characterization and thus provide useful information in predicting therapeutic responses in patients. These laboratory studies have the great advantage that parasites from many different locations or with known resistance profiles can be studied and compared. It is argued below that if the relationship between the standard *in vitro* susceptibility measures (50% inhibitory concentrations [IC_50_], IC_90_, etc.) and *in vivo* PK-PD responses in patients with malaria could be characterized, then this would facilitate dose finding.

## ANTIMALARIAL PHARMACOKINETIC-PHARMACODYNAMIC RELATIONSHIPS

**Fig 3:**
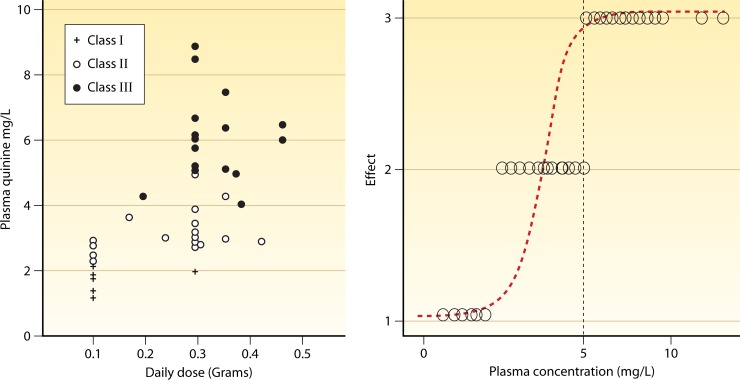
Dose-response relationships obtained between the years 1945 and 1946 for quinine in blood-induced vivax malaria (McCoy strain) in volunteers (38). Plasma concentrations after protein precipitation were measured spectrophotometrically, which overestimates parent compound concentrations. The left box shows the variable relationships between dose and mean plasma concentrations, and the right graph shows the concentration-effect relationship divided into three effect measures: class I, no certain effect; class II, temporary suppression of parasitemia and/or fever; class III, “permanent” effect, i.e., absence of parasitemia for 14 days.

Some of the best research on antimalarial PK-PD relationships came from the period of intense antimalarial drug investigation in the United States during and shortly after the Second World War (Fig. 3). Studies were conducted to determine the optimum dosing strategies for mepacrine (atebrine, quinacrine), the Cinchona alkaloids, and both the 4- and 8-aminoquinolines (37–39). Pharmacokinetic analysis had yet to be invented, and methods for quantitation of drugs in serum or plasma were in their infancy, but the spectrophotometric assays that were conducted still provided valuable information. Relatively large numbers of nonimmune adult male volunteers artificially infected with single “strains” of P. falciparum or P. vivax received different dose regimens, serum levels were measured, and therapeutic responses were assessed. This research provided dose-response or concentration-effect relationships and led to the mepacrine loading-dose regimen, characterization of the comparative antimalarial effects of the four main Cinchona alkaloids (quinine, quinidine, cinchonine, and cinchonidine), and development of the standard dosing regimen for chloroquine (one of the few antimalarial dose recommendations which has stood the test of time). This was still the era of malaria therapy, and the war had focused military attention on malaria. Such volunteer studies are no longer possible today. Since that time, PK-PD relationships have been inferred mainly from clinical studies of antimalarial treatment (8, 9, 40–43).

### (i) PK-PD correlates.

**Fig 4:**
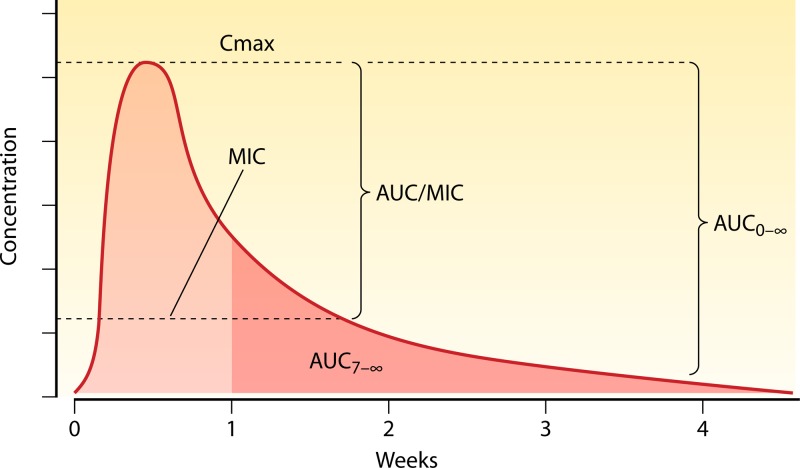
Plasma or blood concentration profile of a slowly eliminated antimalarial drug showing an arbitrary MIC. The AUC is the area under the curve, and Cmax is the maximum concentration in blood or plasma. AUC from 7 days to infinity is shown in darker pink. Blood concentrations are increasingly measured on day 7 in therapeutic assessments of slowly eliminated antimalarials (49).

**Fig 5:**
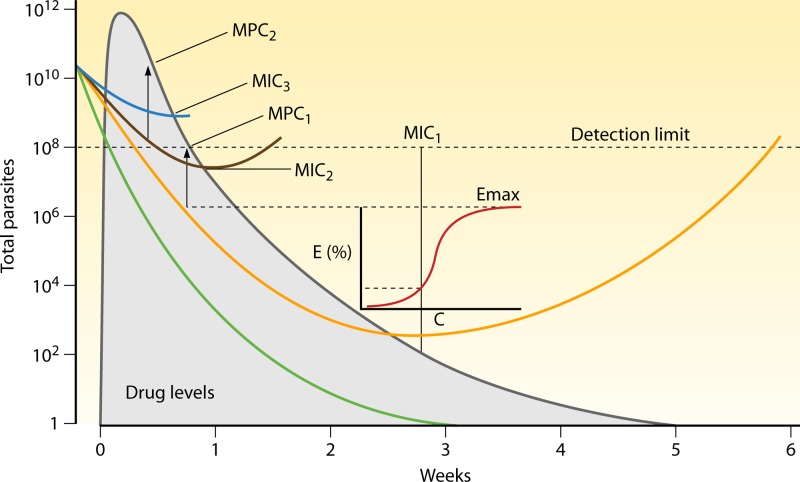
Different therapeutic responses to a slowly eliminated antimalarial drug in a malaria infection of 10^10^ parasites (parasite density, ∼2,000/μl). The blood concentration profile in gray is shown in the background. Parasitological responses range from fully sensitive (green) to highly resistant (blue). Each response is associated with a different level of susceptibility and thus a different MIC and MPC (arrows pointing to concentration profile). The inset represents the concentration-effect relationship for the lowest level of resistance (resulting in a late failure), showing corresponding points for the MIC and MPC (orange curve).

Studies of PK-PD relationships for antibacterial effects have shown that for some antibiotics (those with steep concentration-effect relationships and without postantibiotic effects), bacterial killing is dependent on the duration for which the antibiotic exceeds the MIC for the bacterial population (“time above MIC”). For other antibiotics (where concentrations achieved with current regimens remain on the steep part of the concentration-effect relationship), it is the maximum concentration achieved (*C*_max_) or the related area under the plasma concentration-time curve (AUC) that is the best correlate of bacterial killing (Fig. 4). These PK variables are all interrelated (i.e., the higher the *C*_max_, the larger the AUC and the longer the time above the MIC). With some adjustments, these PK measures can be applied to antimalarial effects (32), although correlates with parasite killing have not been established for most antimalarial drugs. Whereas most bacteria replicate every 20 to 40 min, asexual malaria parasites infecting humans replicate every 1 to 3 days. Symptomatic infections usually comprise one predominant brood of malaria parasites, but multiple genotypes are often present—particularly in higher-transmission settings—and so within one host there may be subpopulations with different drug susceptibilities (and also different stages of asexual development). The lowest blood, plasma, or free plasma concentration which produces the maximum PRR is the MPC (Fig. 5). These PK-PD variables reflect the antiparasitic effects of the antimalarial drug and host immunity and so are specific for an individual and that individual's infection. Innate host-defense mechanisms and acquired immune responses contribute significantly to therapeutic responses—effectively shifting dose-response curves to the left. The contribution of the host immune response, which may be significant even in previously nonimmune patients (44), has not been well characterized.

With current dosing for all antimalarial drugs except the artemisinin derivatives, drug elimination is sufficiently slow that the antimalarial effects of a treatment persist for longer than one asexual cycle (8, 22, 32, 45). Indeed, many antimalarials have terminal elimination half-lives (*t*_1/2_β) of several days or weeks. In order to cure the blood-stage infection in a nonimmune patient, antimalarial concentrations in blood (free plasma concentrations) must exceed the MIC for the infecting parasites until the last parasite is killed (8, 22, 23, 32, 45). The higher the initial parasite burden, the longer this takes. With host immunity, cure of malaria may be achieved even if drug levels fall below the MIC before complete elimination of all parasites (44). Thus, the time above the MIC is an important PK determinant of therapeutic outcome, although the AUC above the MIC is also relevant, as the rate of parasite killing is determined by the concentration-effect relationship above the MIC for the infecting parasites and by the antimalarial concentration profile in the treated patient.

Assuming that the parasites are exposed to the antimalarial drug at a sensitive stage, what duration of exposure in a single asexual life cycle is necessary for maximum effect? For sensitive parasites, it appears that up to 4 h of exposure is required (31), although for some drugs less time is needed. For artemisinin derivatives, killing is generally very rapid, which explains why artemisinin derivatives may be given daily despite their rapid elimination (32). Thus, two factors are important determinants of therapeutic responses: the maximum effect (i.e., maximum PRR) and the initial parasitemia. These determine how long drug levels must exceed the MIC in order to remove all parasites from the body. For rapidly eliminated drugs with PRR values ≥ 10^3^/cycle in nonimmune patients, 7-day regimens (which cover 4 asexual cycles) are necessary. Maximum PRR values range from approximately 10,000/cycle for artemisinins against sensitive parasites down to ∼10/cycle for antimalarial antibiotics (32). All other factors being equal, the increase in the time necessary to eliminate all parasites from the body (Δ time [in hours]) for each increment in initial parasite count is given by the following equation:

(3) $$\Delta \;time\;\left(hrs\right)=\Delta \;{L\mathrm{og}}_{e}\;parasite\;count/k_{p}$$

where *k_p_* represents the parasitemia elimination rate constant derived from the slope of the linear decline in Log_*e*_ parasite counts (per hour) following the start of treatment.

In reality, the other factors are seldom equal. High parasitemia levels reflect the particular patient's inability to control the particular infection, and so the host contribution to parasite clearance may be substantially less in these patients than in most patients with lower levels of parasitemia (46). As a consequence of both parasite numbers and inadequate host defense, high parasite counts (reflecting high parasite burdens) are particular risk factors for treatment failure (46).

**Fig 6:**
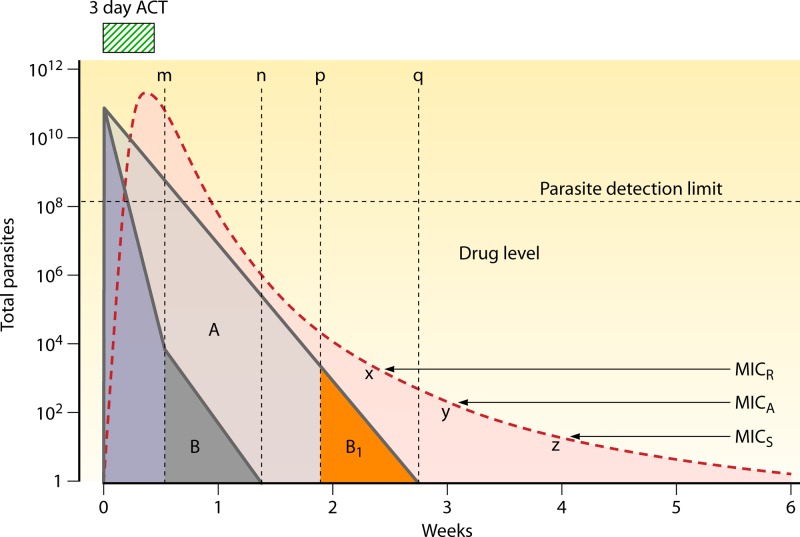
The pharmacokinetic-pharmacodynamic profile of artemisinin combination treatment (ACT) (33). The individual patient parasite burden (approximating 20,000 parasites/μl, corresponding to a total of approximately 10^11^ parasites in an adult) is shown on the vertical axis in a logarithmic scale, and the profile of a slowly eliminated drug's concentrations (illustrated by a single dose of mefloquine) is shown as a red dashed line. The parasites exposed to the antimalarial drugs are shown as triangles. Their areas correspond to total numbers in the blood. The artemisinin component of the treatment is given for 3 days, which covers two asexual cycles. This reduces the parasite burden 100,000,000-fold, which leaves approximately 10,000 parasites (dark gray triangle B) for residual concentrations of mefloquine (from point m to point n) to remove. If no artesunate had been given, the mefloquine would have reduced the parasite burden more slowly (light purple large triangle), and the parasites corresponding to B (i.e., B1) would have been exposed to lower mefloquine concentrations (from point p to point q; orange triangle). In this example, these concentrations would be insufficient to inhibit growth of the most resistant parasites prevalent (MIC; MIC_R_) and so, whereas the ACT would cure all infections provided these blood concentrations were achieved, there would be treatment failures with mefloquine monotherapy. MIC_A_ and MIC_S_ refer to the average and most sensitive MICs, respectively. The time from point x to point z on the mefloquine elimination curve represents the window of selection (circa 16 days in this example) during which newly acquired infections with sensitive parasites cannot establish themselves whereas resistant parasites can.

**Fig 7:**
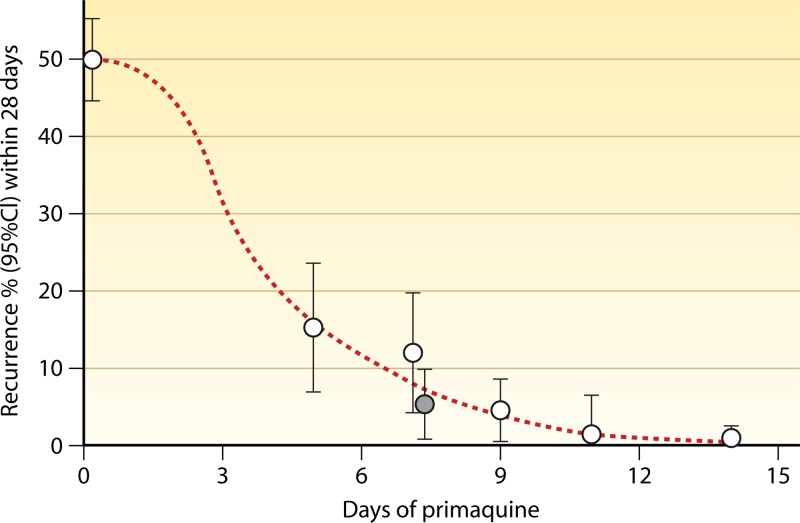
Recurrence rates of P. vivax malaria in adult Thai patients following artesunate treatment (given for 5 or 7 days) combined with different durations of primaquine treatment (30 mg base per day; gray circle, 60 mg per day) (57). These were all assumed to represent relapses, as the artesunate regimen is curative and there was no re-exposure. CI, confidence interval.

While blood concentrations exceed the MPC, a fixed fraction of the infecting parasites are killed each asexual cycle (i.e., parasite killing is a first-order process). This assertion is supported by detailed parasite clearance studies (47) and is consistent with temporal patterns of recrudescent infection. Infections treated with artemisinin derivatives are an exception. Following artemisinin exposure, a small subpopulation of asexual parasites may become “dormant” and thus temporarily refractory to treatment (48). These dormant parasites may give rise to a later (drug-sensitive) recrudescence. As initial blood concentrations for slowly eliminated antimalarials are usually considerably above the MPC and vary with disease-related changes in absorption rates and distribution volumes, it has been suggested that the area under the blood or plasma concentration curve affecting the fourth and subsequent asexual parasite cycles may be a useful predictor of the therapeutic response (cure or recrudescence) in malaria (49). As most patients are seen 1 week after starting antimalarial treatment in therapeutic assessments, the day 7 antimalarial drug concentration, a simple surrogate for the AUC from day 7 to infinity (AUC_7-∞_), is increasingly being measured (Fig. 4). Nearly all antimalarial drugs are in their terminal elimination phase by 1 week after starting drug administration such that the day 7 concentration and the AUC_7-∞_ are linearly related. For artemisinin combination treatments (ACTs), the initial therapeutic response is determined mainly by the artemisinin component which is given for 3 days, covering two asexual parasite cycles. The partner drug is then responsible for removing the residual parasites (numbering up to 100,000) which remain in the third cycle (32, 50) (Fig. 6). Thus, the AUC_7-∞_ or the day 7 concentration reflects this residual partner drug exposure (49). Later concentrations are also proportional, and may be even better predictors of the therapeutic response (51, 52), but they are obviously lower and may approach the limits of assay detection for small-volume blood samples. There have been relatively few studies in recent years of uncomplicated malaria in which PK variables have been related to therapeutic responses. Studies of PK-PD relationships have been performed for quinine, artemisinins, mefloquine, sulfadoxine-pyrimethamine, chlorproguanil-dapsone, and lumefantrine (4, 8, 9, 35, 40–43, 53–56). There are also some recent data for piperaquine (52). Dose-response studies have also been performed to assess the radical curative efficacy (relapse prevention) of primaquine in tropical frequent-relapse P. vivax infections (57) (Fig. 7) and also to assess the transmission-blocking activity of 8-aminoquinolines (58).

The *in vivo* MIC is the critical determinant of the dose and duration of antimalarial treatment, but it has not been well defined for any antimalarial drug. The MIC is the blood or plasma concentration giving a parasite multiplication rate of 1. As drug levels fall below the MIC, then, in the absence of an effective immune response, parasitemia starts to rise again. A recrudescent infection can be detected by microscopy when parasite densities reach approximately 50/μl. Thus, determination of the MIC requires treatment failure. Model-derived estimates have been proposed for quinine in uncomplicated falciparum malaria (42). The MICs differ between infections and, by definition, increase with resistance (8, 32, 50). It is not clear where the MIC lies with respect to the sigmoid concentration-effect relationships for parasite growth inhibition. As P. falciparum and P. vivax multiply at approximately 10-fold per asexual cycle during the exponential-growth phase, and as the MIC gives a multiplication rate of 1, the *in vitro* IC_90_ for free drug may be a useful approximation of the free drug MIC—but this remains to be established. Figure 5 shows the effects of different levels of resistance with progressively higher MICs. The MIC can be modeled if blood concentrations have been measured in an infection which subsequently recrudesces. Suggestions for prospective determination of the *in vivo* MIC during drug development are provided below.

If overall cure rates for existing treatments are high (>90%), as they should be (1), then very large studies are needed to provide the statistical power necessary to correlate pharmacokinetic variables with treatment outcomes, i.e., to compare values between the few patients who have recrudescent infections and the majority who are cured, after adjusting for relevant covariates such as age (as a surrogate for immunity) and parasitemia (59). Taking multiple blood samples, particularly from young children, in large field studies may be unacceptable or impossible. This has led to two developments—the use of sparse sampling and population PK modeling in antimalarial drug assessments (60) and capillary blood filter paper-based drug analyses. These facilitate inclusive large-scale PK-PD assessments. Relationships between day 7 (or day 14) blood concentrations and cure rates have been characterized for lumefantrine, mefloquine, sulfadoxine, pyrimethamine, and piperaquine (4, 8, 9, 49, 55, 56). This is useful in assessing treatment responses (i.e., differentiating poor adherence or pharmacokinetic factors from resistance as causes of therapeutic failure) and therefore in identifying patient subgroups in need of dose adjustments. More information from clinical trials is needed. To achieve this, the capacity for antimalarial drug measurement needs to be expanded in countries where malaria is endemic so that capillary blood drug measurements on day 7 (or at other times) become a routine part of all antimalarial drug assessments in cases of uncomplicated malaria (25, 26, 49).

## ANTIMALARIAL DOSING

For an antimalarial treatment to be reliably (i.e., >95%) effective, it should, by definition, provide >95% efficacy in a nonimmune population. In areas of high transmission, this refers to the younger children, who bear the brunt of the disease, as older children and adults have acquired higher levels of immunity which contribute substantially to therapeutic responses. Self-cure becomes increasingly likely with increasing immunity (44). Calibrating doses against therapeutic responses in semi-immune patients is a mistake, as it results in systematic underdosing in nonimmune patients (1, 22). In areas of low transmission, patients of any age may become ill. For this reason, the initial phase 2 studies with new antimalarials are best carried out in adults with symptomatic malaria. These are followed by phase 2 pharmacokinetic studies in children with malaria. Cure in a nonimmune patient means that all malaria parasites in the blood have been killed before the drug concentrations in blood have fallen below the MIC. To achieve high cure rates, this must occur for >95% of prevalent parasites.

### (i) Resistance considerations.

Ideally, antimalarial treatment should cure everyone thus treated, so drug regimens must be sufficient to cure infections with all prevalent parasites in those patients with the highest parasite burdens. As treatment failure is usually necessary for selection and spread of resistance to occur, it is important that dose regimens are sufficient to ensure very high cure rates (1, 3, 22). It has been argued that the initial deployment of a relatively low dose of mefloquine (15 mg/kg of body weight) initially provided a powerful selective advantage to resistant parasites, whereas use of a higher dose initially (25 mg/kg) would still have cured many of the emerging resistant parasites, thereby reducing the extent of the selective advantage and delaying the onset of resistance (8). Once resistance is selected and spreads, a new set of PK-PD relations pertain. The rate at which resistance spreads depends on the proportion of treatment failures.

A dose (either as a single drug or a combination) that achieves cure in 95% of treated patients might be considered most cost-effective and is a minimum requirement for a new treatment. However, it may be prudent in terms of overall benefit to aim for a higher dose range and thus a higher cure rate if possible. For some drugs (e.g., antifols), resistance develops stepwise in a predictable sequence. Emergence of resistance should be slowed if the chosen dose regimen is also effective against parasites with low-level resistance (8, 41). The concept of mutant prevention, which has been well developed in antibacterial dosing (61), is readily applicable to antimalarial dosing. A dose regimen that ensured that blood concentrations would be sufficient to cure ≥95% of patients infected with parasites with low-level resistance would, by definition, result in a higher total dose. Of course, this might result in greater toxicity and prohibitive costs, but it would be an option worth considering. In contrast, if the first level of resistance encountered is extremely high (e.g., as in the case of the cytochrome B mutations conferring atovaquone resistance which result in approximately 10,000-fold reductions in susceptibility), then there is no point in recommending a total dose higher than that necessary to cure >95% of patients infected with “wild-type” parasites. Fortunately, information on the ease of developing resistance and the level of resistance developed now usually becomes available during drug development, at least for Plasmodium falciparum (18). In considering the economics of drug development, cost-benefit (anticipating future trends) rather than cost-effectiveness (dealing only with the present) analyses may be more appropriate in determining dosing.

While these arguments favor higher dosing as a resistance prevention strategy, there is an alternative view. It has been argued, on the basis of artificially balanced mixed infection studies in rodents, that low doses should be deployed intentionally in order to reduce the fitness disadvantage that drug-sensitive parasites have in the presence of antimalarial drugs and to exploit the competition between different genotypes that limits individual growth (62). Drug-sensitive parasites are considered to provide protection against resistance because in the absence of drugs they are usually fitter and thus outcompete resistant parasites in direct competition. Clearly, this would be in the context of a quasi-steady state of transmission rates sufficiently high that mixed asymptomatic infections would be common and not in one of successful malaria control with declining prevalence. The relevance of these experimental observations in an artificial rodent model of a potential population benefit is uncertain, while there is certainly no doubt that, for an individual patient, underdosing is very dangerous.

### (ii) Dose-response relationships for artemisinin and its derivatives.

Dose-response or concentration-effect relationships can be assessed using parasite clearance rates for any drug which accelerates parasite clearance. A reliably effective antimalarial which does not accelerate ring-form clearance can be given to all patients as a “reference standard” and various doses of the test drug added (63). This ensures that the patient receives an effective treatment. Drugs such as mefloquine or atovaquone-proguanil have few interactions and can be used as the reference standard. Shortening of parasite clearance duration measures can then be plotted against pharmacokinetic variables. Artemisinins are essential components of current antimalarial treatment regimens. Most dose assessments have been done with oral or rectal administration of artesunate (13, 64, 65). Using shortening of parasite clearance as the effect (PD) measure, studies conducted over 10 years ago in Thailand suggested that a 2-mg/kg oral dose of artesunate was the lowest that produced a maximum effect (64). Given the large interindividual variance in concentration profiles, a 4-mg/kg target oral artesunate dose was chosen for ACTs (22). As oral artesunate has approximately 60% bioavailability, the 4-mg/kg oral dose corresponds with the currently recommended 2.4-mg/kg parenteral dose. It is notable that oral doses of artemether (in artemether-lumefantrine) and dihydroartemisinin (in dihydroartemisinin-piperaquine) are substantially lower than this. With respect to *in vitro* antimalarial activity, dihydroartemisinin (molecular weight [MW], 284.4) is equivalent in molar terms to artesunate (MW, 384.4), but in weight terms there is a 35% difference in activities. Artemether is less active *in vitro* than artesunate, although after oral administration approximately two-thirds of the total antimalarial activity is provided by its metabolite dihydroartemisinin (9). Recent data from the Western border of Thailand, where artemisinin susceptibility has declined in recent years, support this 4-mg/kg target oral dose, as a concentration-effect relationship was evident for a 2-mg/kg but not for a 4-mg/kg oral dose of artesunate (66, 67). This indicates that in some patients who received 2 mg/kg, the concentrations of artesunate and its biologically active metabolite dihydroartemisinin did not have a maximal effect (in contrast to the situation 10 years earlier) (64).

If the concentration-effect relationship is not defined, then it is difficult to know whether or not a chosen dose is satisfactory. This dilemma confronted the development of a rectal artesunate formulation for prereferral treatment of severe malaria. To determine whether any patients were receiving insufficient drug, plasma concentrations of artesunate and DHA were measured in patients receiving rectal artesunate and compared with measures of parasite clearance (63). Levels of parasite susceptibility at the time of development differed little, so if some patients had not been receiving sufficient artesunate then there would have been a cluster of patients with slower parasite clearance and lower drug levels. This was not found, which suggested that the dose was sufficient (but did not indicate whether the dose could have been reduced safely).

### (iii) Dosing of individual components in combinations.

Before evaluation of the PK-PD properties of a combination, the PK-PD relationships for the individual drugs should be determined *in vivo* (during phase 2) against well-characterized parasites in acute malaria infections and optimum doses chosen. An evidence-based dose regimen can then be used in phase 3. If possible, the two drugs should be combined at doses which are individually curative. Clearly, for currently recommended first-line ACTs, and for atovaquone-proguanil treatments, this is not the case. In ACTs, the artemisinin component is given for 3 days (and, given alone, provides only an approximate 20% cure rate in nonimmune patients). The slowly eliminated partner drug doses have been chosen at doses which should provide high cure rates in sensitive infections. For piperaquine, amodiaquine, and lumefantrine, this requires three-day dosing in order to load sufficiently to provide therapeutic concentrations (i.e., >MIC) for long enough to eliminate all the parasites. In combinations in which one component is eliminated much more rapidly than the other (such as ACTs), it would seem prudent to aim initially, based on PK-PD studies, for doses of the more slowly eliminated partner drug which are curative. PK-PD studies can then be conducted with the combination to characterize any synergy (or antagonism) with the combination and thus characterize whether the combination dose predicted from the individual drug-dose-finding studies requires adjustment.

### (iv) Severe malaria.

**Fig 8:**
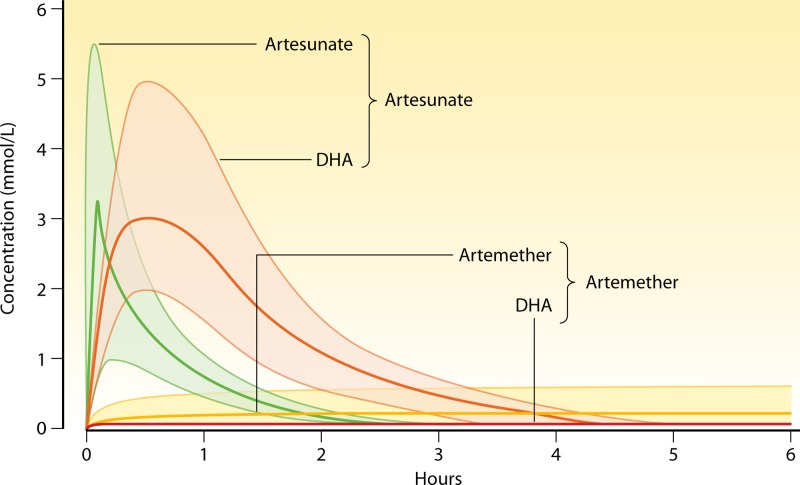
Median and range plasma concentrations of artesunate (green) and artemether (yellow) and their common biologically active metabolite dihydroartemisinin (red) after intramuscular injection measured during the treatment of adult Vietnamese patients with severe falciparum malaria (70).

In cases of severe malaria, antimalarial drug treatments must aim to kill the infecting parasites as quickly as possible (there is no evidence that this is harmful and plenty that it is beneficial) (15, 16, 68). Thus, dose optimization for parenteral antimalarials should concentrate firmly on the initial doses. Ideally, antimalarials should be given intravenously to ensure instant bioavailability, but if this is not possible, intramuscular or rectal routes may be effective. Only three drug classes have ever been used widely to treat severe falciparum malaria: the Cinchona alkaloids, the 4-aminoquinolines, and the artemisinin derivatives. All may be given by the intravenous, intramuscular, and, in suitable formulations, rectal routes. Chloroquine can also be given subcutaneously (69). Artesunate has been shown clearly to be the best treatment for severe malaria in recent very large randomized controlled trials (15, 16). Cinchona alkaloids and 4-aminoquinolines have significant cardiovascular effects and so must be given by rate-controlled infusions and never by intravenous injection (as such administration can result in lethal hypotension) (3, 12). PK-PD assessments in severe malaria have led to dose regimen changes for each of these antimalarials. For quinine, use of a loading dose (20 mg salt/kg) of twice the maintenance dose (10 mg/kg) in Thai adult patients with cerebral malaria shortened the mean time for plasma concentrations to exceed 10 mg/liter (an approximate MPC) from 20.5 h to less than 4 h. This resulted in a halving of the mean parasite clearance time from 103 to 55 h (12). Peak concentrations occurred earlier in the course of treatment but were no different from those seen without a loading dose, so this simple dose adjustment provided benefit without harm. Chloroquine has complex pharmacokinetic properties, with an enormous total apparent volume of distribution and a terminal elimination half-life of approximately 1 month but a relatively small central compartment. Blood concentration profiles and initial therapeutic responses are therefore determined primarily by absorption and distribution rather than by elimination (69). Rapid absorption of chloroquine from intramuscular or subcutaneous injections may result in lethal hypotension. Simple dose adjustments (reducing individual intramuscular or subcutaneous doses from 5 mg base/kg to a maximum of 3.5 mg base/kg) reduced harm without reducing benefit (69). Chloroquine efficacy has since fallen as a result of increased resistance, and the drug is no longer used to treat severe malaria. Artemether and artemotil are oil-based artemisinin-derivative formulations which can be given only by intramuscular injection. Absorption is erratic and may be very poor in cases of severe malaria (70, 71) (Fig. 8). Artesunate given by intravenous or intramuscular routes achieves therapeutic concentrations of parent drug and the common biologically active metabolite dihydroartemisinin much more rapidly and reliably (62, 64). As a consequence, despite similar levels of parasiticidal activity, artesunate is superior to artemether in the treatment of severe malaria (72).

Each of the three drug classes that have been used in severe malaria has very different pharmacokinetic properties and a different stage specificity of action; the artemisinin derivatives kill circulating ring-stage parasites whereas quinine does not (30–32, 73, 74). Chloroquine occupies an intermediate position, with some effect on rings but much less than that seen with the artemisinins (30, 31, 74). All are probably equally effective against sequestered forms (30, 31, 73, 74). The rapid killing of circulating ring forms which prevents their maturation and sequestration explains why artesunate substantially reduces mortality in severe falciparum malaria compared with quinine (3, 15, 16). The pharmacodynamic measure or “readout” of this unique activity is acceleration of parasite clearance (64). The superiority of artesunate over artemether in clinical outcomes is paralleled by more rapid parasitological responses (72). In turn, artemether is superior to quinine in terms of both clinical outcomes and parasite clearance times (68). The benefits of artemisinin derivatives compared to quinine in randomized controlled trials conducted in patients with severe malaria in Asia have been greater than those in Africa (15, 16) despite generally similar pharmacokinetic profiles in African children and Asian adults (13, 17, 65, 70, 71). While the differences were initially attributed to the greater susceptibility of P. falciparum in Africa, to the more rapid time course of disease, and even to differences in pathological processes, recent studies suggest a more prosaic explanation, i.e., misdiagnosis in a significant proportion of African children considered to have severe malaria (75, 76). It is very difficult to distinguish sepsis from severe malaria clinically, and the two conditions commonly occur together.

Very few trials in severe malaria have been powered to detect mortality differences. Whether any of the clinical or laboratory surrogates of therapeutic response in survivors which are continuous variables, such as times to regain consciousness, sit, eat, and stand, fever clearance, resolution of acid-base disturbances (rise in standard bicarbonate concentration [sHCO_3_], fall in lactate level), rates of resolution of renal impairment in adults, or attenuation of the fall in hematocrit, are good correlates of mortality benefits remains to be established. In general, the laboratory variables (in particular, measures of acidosis) have performed better than the clinical measures in clinical trials. In the randomized controlled comparisons of artesunate or artemether versus quinine, which are by far the largest trials ever performed in severe malaria, serial disease severity measures were often worse in the patients receiving artemisinin derivatives despite the substantially lower mortality associated with these treatments (1, 15, 16, 68, 72). This apparent paradox is probably explained by the following: patients whose lives are saved by a better treatment (i.e., artesunate compared with quinine) are inevitably the most seriously ill. If they survive, they, and their laboratory measurements, take longer to recover. As a result, measures of recovery in survivors can be better in the group treated with the less effective drug.

## NEW ANTIMALARIAL DRUGS

Several antimalarial drugs are in the late stages of development or have entered clinical trials. Arterolane (Rbx11160) is registered in India, and OZ439 and the spiroindolone KAE609 are in phase 2 studies. As most currently available antimalarials are well tolerated, these new drugs will have to match this good acceptability and toxicity profile while being highly efficacious. There is now a critical window of opportunity early in clinical development, before the new drugs are combined with partner antimalarials, in which to identify optimum doses (20). Dose finding should be based upon characterization of concentration-effect relationships for both efficacy and toxicity. The target is to remove up to 10^13^ (usually between 10^8^ and 10^12^) parasites. The ideal treatment would provide a reliable single-dose cure, but this requires providing reliably PRR values close to >10^3^/cycle for 9 days (5 cycles) or >10^4^/cycle for 7 days (covering 4 cycles) against all prevalent parasites.

Optimized dosing should limit the emergence and spread of resistance and maximize cost-benefit (30). The initial clinical steps are very important, as it would be difficult to revisit dose-finding studies once phase 3 studies are under way, particularly if the new drug is locked in a fixed combination with another drug. When a new antimalarial drug enters phase 2 studies, it is first necessary to confirm antimalarial activity *in vivo* in humans (a proof of concept). The initial dose will have been chosen on the basis of the doses used in the phase 1 studies, which in turn will have been extrapolated from animal and *in vitro* studies. If this is a critical decision point (“go/no go”), then a predicted “high” dose will probably be chosen for the “confirmation of antimalarial activity *in vivo* in humans study” to avoid a false-negative result and unwarranted discontinuation of the drug's development. Volunteer patients in phase 2 studies need to be fully informed of the experimental nature of the study they may participate in, the risks involved, and the absolute need to stay under medical supervision until they have cleared parasitemia and are clinically well. Physicians and nurses need to know the risks and to have low thresholds for providing known effective rescue treatment in cases of clinical deterioration.

### (i) Confirmation of antimalarial activity *in vivo* in humans.

Initial phase 2 antimalarial studies with new antimalarial compounds should always be performed in nonpregnant adults. Falciparum malaria is inevitably the primary focus, but it is a dangerous disease. Despite the extensive experience with artificially induced malaria in humans, ethics review boards and investigators may be reluctant to take the risk of conducting experiments with an untried medicine in symptomatic patients with falciparum malaria even in intensively supported facilities. There are three alternative approaches to conducting initial “confirmation of antimalarial activity *in vivo* in humans” phase 2 studies which are safer than first use in patients with symptomatic falciparum malaria.

1. Instead of falciparum malaria, the less dangerous P. vivax malaria could be studied initially. This would provide *in vivo* data and useful information on PK-PD relationships in disease. P. vivax malaria also has the advantage that it does not sequester, and so, unlike P. falciparum infections, peripheral blood parasite counts correlate closely with the infecting parasite burden, making initial interpretation of parasitological responses more straightforward.
2. The first volunteers could be adults with asymptomatic falciparum malaria. However, asymptomatic infections are generally highly sensitive to any intervention because of the effective immune responses which controlled the infection in the first place. This approach is therefore likely to overestimate the drug effect substantially. Furthermore, parasitemia levels are always low in asymptomatic adults, providing relatively little information on the kinetics of parasite clearance. Finally, the pharmacokinetic properties of the new drug would not reflect acute illness effects.
3. Volunteers can be artificially infected with a known drug-sensitive malarial “strain” and the evaluation conducted entirely at parasitemia levels below the pyrogenic density using very sensitive quantitative PCR (qPCR) methods (21, 77–79). Similar approaches have been taken in vaccine evaluations (77, 80, 81). This assessment should reflect antimalarial responses in symptomatic malaria, although any disease effects on antimalarial PKs or PK-PD responses would be minimized, and it does rely heavily on accurate measurement at very low densities. The accuracy of the sensitive detection methods in characterizing parasite clearance kinetics needs to be established further, but this is certainly a very promising approach for “confirmation of antimalarial activity *in vivo* in humans.”

Whichever approach is chosen, frequent antimalarial blood concentration measurements should be made in an attempt to characterize the PK-PD responses, as this would help inform design of the definitive dose-finding studies. Once confirmation of antimalarial activity *in vivo* in humans is satisfactorily obtained, the conventional approach to dose finding is to reduce doses steadily in small groups of patients until there is clear evidence of a reduced antimalarial effect. This has meant comparing recrudescence rates at different doses—a costly and time-consuming process. Relatively large numbers are needed for adequate precision, so estimates are inevitably imprecise (see below). Even then, the lowest dose providing “satisfactory cure rates” applies to a particular set of patients (usually adults) with a particular set of infecting parasites.

### (ii) Dose and duration.

There are two linked components used in assessing treatment responses—the initial response (reflecting drug effects over one or two asexual parasite life cycles) and the overall curative efficacy (reflecting drug effects over three or more asexual parasite life cycles) (22, 23). The initial responses should be maximum (maximum PRR for that antimalarial drug) in any dose regimen—but curative efficacy depends also on the duration of exposure. This is illustrated by the artemisinins, where 5-day regimens are required for cure of P. vivax infections and 7-day regimens for P. falciparum and yet ACTs are prescribed in 3-day regimens. These provide maximum parasite killing effects during two asexual cycles but still leave up to 100,000 parasites for the partner drug to remove. All finally recommended treatments should ideally provide antimalarial concentrations which exceed the MPC for ≥4 parasite life cycles (this is necessary to cure infections of >10^12^ parasites with a PRR of 10^3^ per cycle) (22, 23, 32, 33).

### (iii) Dose-response assessments based upon initial antimalarial responses.

With new or experimental treatments, clear clinical and parasitological criteria for treatment failure and patient rescue with an effective antimalarial treatment should be predefined (e.g., if parasitemia does not fall by >75% in 24 h). Frequent sampling for antimalarial drug concentration profiling is needed so that confident assessments of key PK variables can be made. If the PD readout is slowing of parasite clearance (assessed as parasite clearance rate or half-life [33, 34] or by a more complex measure incorporating lag phase and slope), then a sufficient number of patients must be studied to be confident of submaximum effects. There is likely to be considerable interindividual variance in blood concentration profiles as well as PD responses, so, when doses are reduced, it is likely that some patients in a group show submaximal responses and some do not. An additional complexity is that slowly eliminated antimalarial drugs with large apparent distribution volumes require loading in multiple doses, so the initial concentration profile is determined primarily by distribution (8, 9, 69). Thus, parasite clearance after the initial dose may underestimate the effects obtained by higher concentrations after loading. It may be difficult to derive useful dose-response relationships from single doses in such cases.

If the volunteer patient has falciparum malaria and parasite clearance is the primary PD readout (e.g., evaluation of a drug with ring-stage activity such as a new peroxide), then, as described earlier, a second drug such as mefloquine or atovaquone-proguanil may be given simultaneously with or shortly after administration of the first drug to ensure cure (64). Although the contributions of the two drugs cannot be separated, the majority of the initial changes in parasitemia result from the peroxide component, allowing PK-PD assessment. Even for drugs which do not affect ring stages, measures of parasite clearance can still be used in an attempt to define the concentration-effect relationship, although there is more interindividual variance in responses because of the variable parasite stage of development at presentation. This large interindividual variance in parasite clearance responses will probably necessitate relatively large sample sizes for adequate precision using this approach to dose finding.

### (iv) Dose-response assessments based upon overall curative efficacy.

For slowly eliminated drugs, the issue is to determine how much drug in total is needed to cure reliably; for rapidly eliminated drugs, it is to determine for how many days the drug should be given. If there is capacity-limited (saturable) absorption of a drug (e.g., lumefantrine), then dose spacing is also a consideration. In a conventional approach to dose finding, the doses need to be reduced early in the phase 2 studies to determine which concentration profiles are necessary for cure. This inevitably means observing recrudescences—although these may be detected and treated before they become symptomatic if sensitive qPCR parasitemia monitoring is conducted. As new antimalarial drugs should achieve at least 95% efficacy and should no longer be recommended when cure rates fall below 90%, obtaining precision around such high proportions of efficacy requires very large sample sizes (59). Dose finding is rightly not conducted in patients with high parasitemia levels for safety reasons—and yet these are the very patients most likely to fail treatment, and they are an important source of *de novo* resistance (46). Dose regimens which are highly effective at low parasitemia levels may not be curative at higher densities. As a consequence, the doses chosen initially are usually too low because they were optimized for infections with low parasite burdens. In areas of malaria endemicity, patients, commonly children, may present with uncomplicated falciparum malaria and parasite counts well in excess of 200,000/μl (corresponding to >10^12^ parasites in the body), which is considerably higher than that in the entry criteria for dose-finding studies. If there is no other PK-PD characterization, then treating different groups of patients with different doses and recording cure rates is likely to be an imprecise and rather expensive method of dose optimization which results in dose recommendations which are too low.

## DISADVANTAGES OF THE CONVENTIONAL MULTIPLE-DOSE STRATIFIED APPROACH TO DOSE FINDING

(i) If the parasite clearance rate is used as an endpoint:

1. Large variations in antimalarial blood concentrations may confound estimates.
2. For drugs with large distribution volumes and low clearance, loading may require multiple doses, so dose-response relationships estimated from initial concentration profiles may be different from those estimated after loading.
3. Large interpatient variations in parasite clearance result in imprecise estimates.
4. Dangerous complications may arise in undertreated patients.
5. These approaches identify only the MPC, or another PK correlate, but not the MIC.
6. Drug absorption lag times may confound estimates.
7. If a second drug is given concomitantly for safety reasons, then individual drug effects cannot be separated reliably.

(ii) If the cure rate is used as an endpoint:

1. Large sample sizes are required as groups, not individuals, are compared.
2. Dangerous complications may arise in undertreated patients.
3. PK correlates may be inaccurate; this approach does not identify the MIC or MPC.
4. Patients with high parasite burdens have the highest risk of treatment failure, and yet they are systematically excluded from evaluations of drugs in development.

In both cases, the relationship between the *in vitro* susceptibility of the infecting parasites and the therapeutic response is not well characterized.

## ALTERNATIVE APPROACHES TO DOSE FINDING

An alternative approach to dose finding is to define the *in vivo* MIC as the primary objective of phase 2 studies. If the *in vivo* MIC can be defined, then comparison of *in vivo* MIC (and, hopefully, MPC) estimates with *in vitro* parasite susceptibility measurements from the same malaria infections would allow calibration of the large body of *in vitro* data on drug susceptibility that can be generated rapidly from parasite isolates already obtained from across the world. The inhibitory concentration *in vitro* which corresponds with the MIC and MPC can be identified and the corresponding values for the most resistant parasite isolates extrapolated. These assessments of variance in parasite susceptibility (PD) can then be melded with estimates of PK variance from population pharmacokinetic studies conducted in different patient populations to predict the dosing regimens necessary to ensure cure if the most resistant natural isolates infected the patients with lowest drug exposures.

### (i) Combining *in vitro* and *in vivo* data to inform dose finding.

New antimalarial drugs should be evaluated in *in vitro* susceptibility tests performed with a large number of P. falciparum (and, increasingly, P. vivax) isolates across different geographic regions (18). This provides data to describe the natural variation in parasite susceptibility. Antimalarial compounds are unlikely to have reached the clinical development phase if there is marked variability in parasite susceptibility or if high-grade resistance is very readily selected. It is essential for this objective that standardized methods of conducting and analyzing *in vitro* susceptibility tests are agreed upon (27) so that results from different laboratories can be compared. Laboratory selection studies should be performed to determine how readily resistance can be induced (18), what degree of resistance can be selected, the underlying molecular mechanisms, and whether or not multiple steps occur in a predictable sequence. Such data have usually been viewed in isolation because of the poorly characterized relationship between *in vivo* and *in vitro* susceptibility. Yet *in vitro* susceptibility information can be obtained rapidly from studies of diverse parasites in diverse geographic locations, thereby providing valuable pharmacodynamic information on the range of natural susceptibilities. Provided the treatments are well tolerated, dose finding aims to find a dose and duration of treatment that will reliably cure infections with the least susceptible parasites at high burdens in patients with no background immunity. Calibrating parasite susceptibility data against *in vivo* responses, and combining these with population pharmacokinetic data in different populations, allows evidence-based prediction of likely therapeutic responses in different patient groups with different pharmacokinetics across a range of parasite burdens.

### (ii) Defining the MIC.

Defining the *in vivo* concentration-effect relationship allows prediction of how much drug is required for treatment. While blood concentrations of an antimalarial drug (or drugs) exceed the MIC in malaria, parasite numbers decline, so a generally efficacious regimen is one that maintains blood concentrations above the MIC in all patients until complete elimination of even the most drug-resistant naturally occurring infection. The MIC is a useful theoretical concept, but its utility in dose finding and other aspects of therapeutic assessment remains to be defined. It is not known how variable it is. As the MIC represents both drug and host effects, we do not know yet how much it is affected by the host response even in nonimmune patients. Host contributions to parasite clearance in malaria reflect the duration and severity of the initial infection and so are likely to be lowest early in acute infections in previously unexposed individuals.

**Fig 9:**
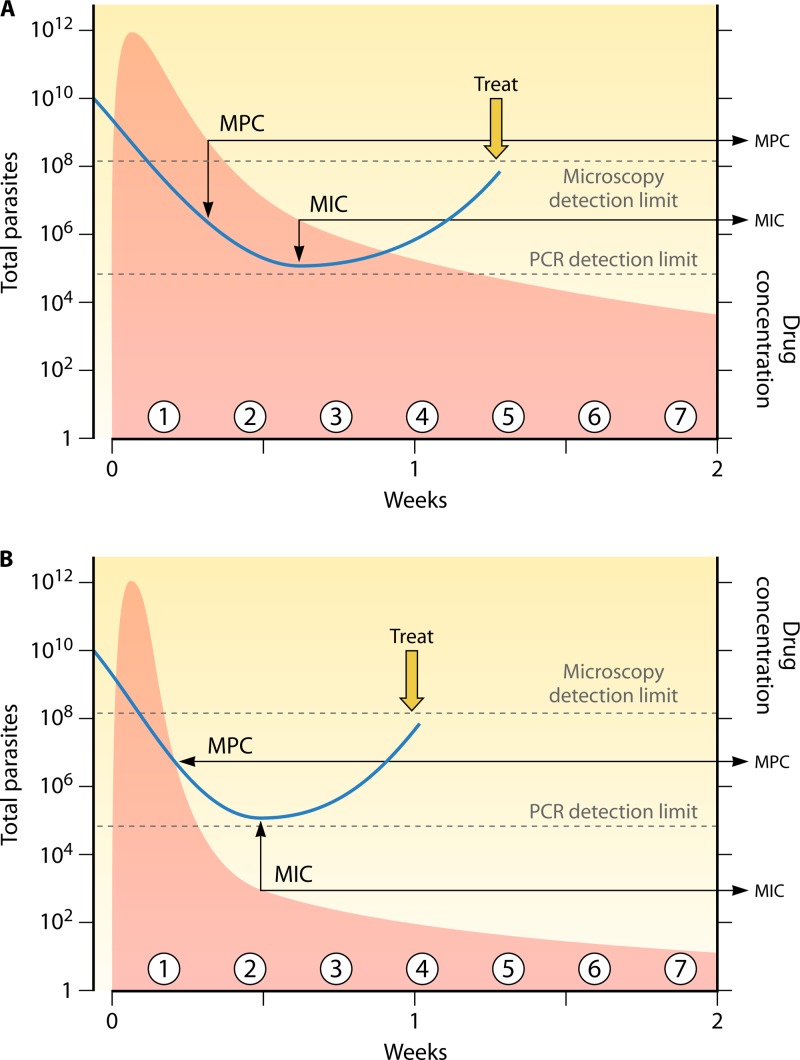
Measuring the MIC. (A) The single dose of slowly eliminated antimalarial under investigation results in fever resolution and clearance of parasitemia as estimated by microscopy. Numbers of total parasites in the body of an adult are shown on the vertical axis. As plasma concentrations (shown in pink) fall below the MPC, the rate of decline of parasite density (blue line), which is now being measured by a sensitive PCR method, is reduced, reaching a plateau between the third and fourth posttreatment asexual cycles (around 1 week). The corresponding plasma concentration at this transient steady state, when the parasite multiplication factor is 1, is the MIC. The level of parasitemia then begins to rise as plasma concentrations fall further. (B) The plasma concentration profile is different, with a rapid initial fall as the drug distributes, followed by a slower elimination phase. Interpretation of the parasitemia plateau, and thus the MIC, is less clear. The dose for MIC estimation is chosen to provide MICs when the drug is in the terminal elimination phase while parasitemia levels can still be quantitated by qPCR.

In theory, the MIC is much easier to estimate for drugs which are slowly eliminated (half lives > asexual cycle length) and do not have prominent distribution phases. This is because the important confounders such as variable stage susceptibility within the asexual cycle, or second-cycle effects, operate within a narrow range of slowly declining blood concentrations so that the estimate is more precise (Fig. 9A). Estimating an *in vivo* MIC against fluctuating drug concentrations, either for a rapidly eliminated drug such as an artemisinin derivative or for a drug for which distribution determines initial concentration profiles and antimalarial effects (such as chloroquine or piperaquine), is more difficult or may not be possible (Fig. 9B).

### (iii) Experimental approach.

Estimation of the MIC requires measurement of blood concentrations (whole blood, plasma, or free plasma levels) and parasite densities at a time when the multiplication rate is 1. This would usually precede by days or weeks a subsequent recrudescence. One approach to characterize the *in vivo* concentration-effect relationship would be to give a single relatively low dose of the new antimalarial compound to volunteer patients during the initial dose-finding studies and then to follow the blood concentrations and the parasite densities frequently using microscopy and then sensitive validated quantitative PCR methods (21, 79–81). As blood concentrations of the antimalarial drug fall below the MPC, the rate of decline in parasitemia (quantitated by serial sensitive qPCR measurements) slows until the parasite density plateaus temporarily and then begins to rise again. The antimalarial drug blood concentrations coinciding with the plateau contain the MIC (Fig. 9). It is apparent that the more rapid the decline in blood concentrations, the broader the estimate of MIC, as it encompasses the concentration range over a two-day cycle; i.e., the precision of the estimate is directly proportional to the elimination half-life. This approach is under consideration for two new antimalarial drugs entering phase 2 studies. With a suitable dose, the MIC should occur after the patient has recovered from the febrile illness and usually when parasite densities have fallen below the level of microscopy detection. Ideally, the initial decline in parasite density should be maximum so the MPC (i.e., the blood concentration when the decline in parasite counts begins to slow) could be estimated as well. Once a dose in an individual patient has been identified which provides MICs in the qPCR-measurable range, this dose can be repeated in further patient volunteers to begin characterizing the distribution of MICs among different infections. Sensitive qPCR methods now allow detection of very low densities (usually ≥10 parasites/ml, which represents sensitivity up to 1,000 times greater than microscopy), so it is possible to follow the subpatent parasitemia levels as they decline and then rise again. The serial microscopy and qPCR estimations of parasitemia should be reported in real time and, once there is clear evidence that parasitemia is rising again, but before illness develops, definitive treatment given promptly. In this way, recurrent illness symptoms should be prevented, and so the volunteer patient would be unaware of the recrudescence. It may not be possible to measure both MPC and MIC directly with the initial doses given, if the parasite densities at MIC fall below the level of qPCR detection (Fig. 9). A major potential confounder in falciparum malaria is slower clearance of gametocytes. This can be assessed by mRNA quantitation, which allows distinction of sexual from asexual forms, but this is less sensitive than DNA detection methods. The simple alternative is to give a single low dose of primaquine (0.25 mg base/kg) in addition to the drug under assessment, as primaquine at this dose has maximum gametocytocidal but negligible asexual-stage activity against P. falciparum and in any case is eliminated rapidly (58). As proof of principle requires showing that parasitemia is reduced (but does not require cure), this single-dose approach could be taken from the outset in dose-finding studies. The initial PK-PD estimates derived from the first doses given could be used to choose the doses required for accurate PK-PD characterization in an iterative process.

When the parasite multiplication factor falls to 1 at a total body parasite biomass of less than approximately 100,000 parasites, then parasite densities cannot be quantitated, and so the MIC cannot be measured directly (although it can still be inferred from modeling) (Fig. 9). The dose required to evaluate the MIC is therefore critical, as too high a dose will drive parasite numbers below the level of detection and too low a dose will not cure the patient initially. For drugs with a marked distribution phase, the MIC is best determined during the terminal elimination phase. This requires choosing a dose sufficient for the MIC to occur during elimination rather than distribution.

## IMPLICATIONS FOR COMBINATION TREATMENTS

It is now generally agreed that in order to prevent the emergence and spread of resistance, antimalarial drugs should be deployed in combinations. How then should the individual components be evaluated, when they will never be deployed as single drugs? The default position often quoted is that each component should be evaluated independently, and the curative dose established, before putting the drugs together in combination. This is not always necessary for rapidly eliminated drugs. Indeed, the results of such an approach may be misleading. For example, artemisinin combination treatments (ACTs) all comprise a noncurative dose regimen of artesunate, artemether, or dihydroartemisinin. The value of assessing cure rates with these drugs (further complicated by drug-specific parasite dormancy) in determining dose regimens in ACTs is dubious. Furthermore, even if drugs have well-matched pharmacokinetic properties and have no PK or PD interactions, the doses of each component in a combination required to produce >95% cure are nearly always less than those of the drugs used alone; i.e., if the drugs have unlinked distribution and elimination pathways and different mechanisms of action, then in >95% of the occasions when concentration profiles of one drug are insufficient to cure, the other drug will be curative. The exact magnitude of the combination advantage depends on the PK-PD distributions for each drug. The alternative PK-PD approach detailed above with assessment of pharmacokinetic interactions and pharmacodynamic synergy and antagonism should obviate costly and time-consuming assessment of individual drug cure rates at different doses.

## CONCLUSIONS

Investing in characterization of antimalarial drug pharmacokinetic-pharmacodynamic relationships will probably improve current antimalarial dose regimens and will certainly increase the likelihood of choosing an optimum regimen for newly introduced antimalarials.
